# FRESH AND LEAN BEEF INTAKE IN RELATION TO FUNCTIONAL LIMITATIONS AMONG U.S. OLDER ADULTS, 2005-2016

**DOI:** 10.1093/geroni/igz038.1766

**Published:** 2019-11-08

**Authors:** Ruopeng An, Sharon Nickols-Richardson, Reginald Alston, Sa Shen, Caitlin Clarke

**Affiliations:** 1 University of Illinois at Urban-Champaign, Champaign, Illinois, United States; 2 University of Illinois at Urbana-Champaign, Champaign, United States

## Abstract

Beef is a key component in the American diet. This study assessed fresh and fresh lean beef intake in relation to functional limitations among U.S. older adults 65 years and older. Logistic regressions were performed on individual-level 24-hour dietary recall and health indicator data (N=6,135) retrieved from 2005–2016 National Health and Nutrition Examination Survey. Approximately 51%, 14%, and 9% of older adults consumed beef, fresh beef, and fresh lean beef, respectively. Daily increase in fresh beef consumption by 1 ounce-equivalent was associated with a reduction in the odds of lower extremity mobility limitation (LEM) by 16% (95% confidence interval=4%–27%), general physical activities limitation by 13% (1%–24%), and any functional limitation by 14% (2%–24%). Daily increase in fresh lean beef consumption by 1 ounce-equivalent was associated with a reduction in the odds of LEM by 22% (7%–34%) and any functional limitation by 15% (1%–28%). No association with activities of daily living, instrumental activities of daily living, or leisure and social activities limitations was identified. In conclusion, preliminary evidence links fresh and fresh lean beef consumption to reduced functional limitation risk. Older beef consumers are encouraged to modestly increase their intakes of fresh and lean beef, rather than total beef, to maximize attributes of functional health associated with beef consumption while concurrently avoiding additional saturated fat and sodium intake. Limitations of this study include measurement errors and cross-sectional study design. Future studies with longitudinal/experimental design are warranted to examine the influence of fresh/lean beef consumption on functional limitations among older adults.

